# Identification of differential expression genes associated with host selection and adaptation between two sibling insect species by transcriptional profile analysis

**DOI:** 10.1186/1471-2164-14-582

**Published:** 2013-08-28

**Authors:** Haichao Li, Hao Zhang, Ruobing Guan, Xuexia Miao

**Affiliations:** 1 Key Laboratory of Insect Developmental and Evolutionary Biology, Institute of Plant Physiology and Ecology, Shanghai Institutes for Biological Sciences, Chinese Academy of Sciences, Shanghai 200032, China; 2 University of Chinese Academy of Sciences, Beijing 100049, China

**Keywords:** Development, Host plant range, Transcripts, Digital gene expression tag profile (DGE-Tag), Sibling species, Differential expression gene, *Helicoverpa armigera*, *Helicoverpa assulta*

## Abstract

**Background:**

Cotton bollworm (*Helicoverpa armigera*) and oriental tobacco budworm (*Helicoverpa assulta*) are noctuid sibling species. Under artificial manipulation, they can mate and produce fertile offspring. As serious agricultural insect pests, cotton bollworms are euryphagous insects, but oriental tobacco budworms are oligophagous insects. To identify the differentially expressed genes that affect host recognition and host adaptation between the two species, we constructed digital gene expression tag profiles for four developmental stages of the two species. High-throughput sequencing results indicated that we have got more than 23 million 17nt clean tags from both species, respectively. The number of unique clean tags was nearly same in both species (approximately 357,000).

**Results:**

According to the gene annotation results, we identified 83 and 68 olfaction related transcripts from *H. armigera* and *H. assulta*, respectively. At the same time, 1137 and 1138 transcripts of digestion enzymes were identified from the two species. Among the olfaction related transcripts, more odorant binding protein and G protein-coupled receptor were identified in *H. armigera* than in *H. assulta*. Among the digestion enzymes, there are more detoxification enzyme, e.g. P450, carboxypeptidase and ATPase in *H. assulta* than in *H. armigera*. These differences partially explain that because of the narrow host plant range of *H. assulta*, more detoxification enzymes would help them increase the food detoxification and utilization efficiency.

**Conclusions:**

This study supplied some differentially expressed genes affecting host selection and adaptation between the two sibling species. These genes will be useful information for studying on the evolution of host plant selection. It also provides some important target genes for insect species-specific control by RNAi technology.

## Background

Cotton bollworm (*Helicoverpa armigera*, Hübner) and oriental tobacco budworm (*Helicoverpa assulta*, Guenée) are two sibling noctuid species of Lepidoptera. They are distributed in almost same region from 50°S to 50°N and from 45°S to 45°N, respectively. *H. armigera* are slightly broader than oriental tobacco budworms [1,2]. In the field, similar external morphology makes them easily confused.

Interestingly, these two species can mate under artificial manipulation and produce offspring. However, when female *H. armigera* mated with male *H. assulta*, the first filial generations are all males [3]. This result further confirms that they are two distinct species [4]. Under natural conditions, because of differences in sex pheromone composition, the two species seldom mate. Their sex pheromones comprise *cis*-11-hexadecenal (Z11-16: Ald) and *cis*-9-hexadecenal (Z9-16: Ald), but the compositions are reversed in the two species. The ratio of these two components is 97:3 in *H. armigera*, but it is 7:93 in *H. assulta*[5-7].

In addition, the host ranges of these two species are significantly different. *H. armigera* is a euryphagous insect whose host range includes 40 families of over 200 different plants. However, *H. assulta* is an oligophagous insect, they are mainly feeding on the plants of the Solanaceae, for example, tobacco and hot pepper [1,8,9]. Although each species have their own preferred host plants, both of them love feed on tobacco and hot pepper [4]. These similarity and difference may be depend on the host plant selection by adult, or depend on the food digestion or detoxification enzymes from larvae. Host plant selection is a complicated and continuous process. The color, odor and shape of the plants will affect the insects choice on host plants, among which odors are a critically important element for the lifestyle and reproduction of an insect species [10]. Accordingly, insects with different feeding habits possess their own specific odor identification and odor-binding proteins [11]. At the same time, enzymes for food digestion and detoxification are also important factors for insect growth and development. These enzymes probably effect on the survival of insects, consequently affecting on the host range of an insect species. Therefore, identifying enzymes related to insect development and feeding habits will benefit to research on insect host range and on insect pest control.

The two sibling species are non-model insects. Their genome sequences are not available till now. To identify differentially expressed genes from *H. armigera* and *H. assulta*, digital gene expression tag (DGE-tag) profile libraries were constructed and sequenced using high throughput second-generation sequencing technology [12,13]. A DGE-tag profile is according to the theory and method of SAGE (serial analysis of gene expression) combined with high-throughput sequencing technology [14]. In this study, eight DGE-tag libraries were constructed and sequenced for four developmental stages (embryo, larva, pupa and adult) of the two species. Differentially expressed transcripts or genes between *H. armigera* and *H. assulta* were analyzed by bioinformatics. Most growth and development related genes have similar expression modes. The differentially expressed genes are mainly focus on olfactory-related genes and enzymes for food detoxification or digestion. Therefore, the two sibling species represent a good model for host plant selection and adaptability. These results also provide valuable data for insect pest control.

## Results and discussion

### The main identifying characteristics of *H. armigera* and *H. assulta*

**Figure 1:**
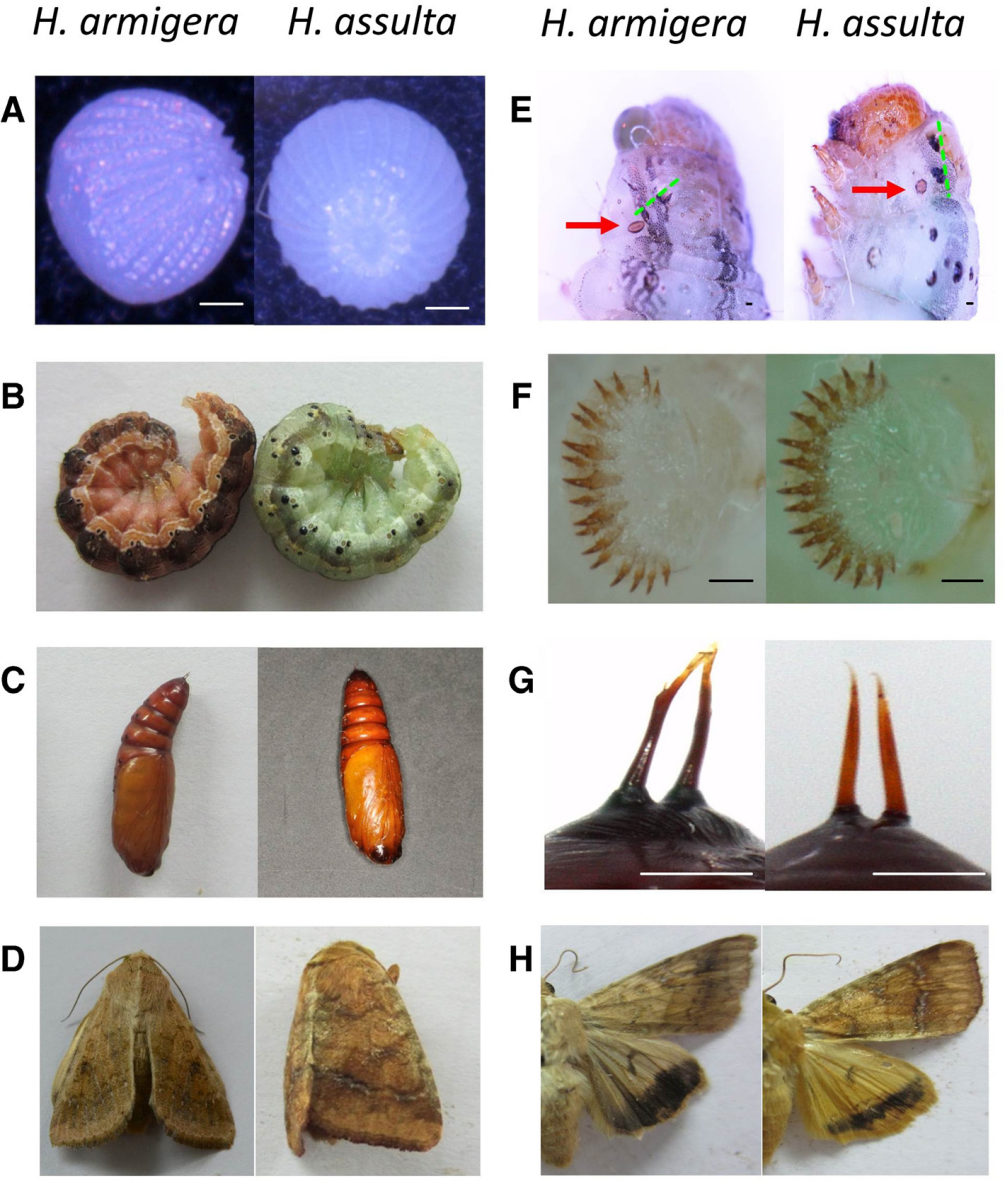
**Main taxonomic characteristics of *H. armigera *and *H. assulta*. (A) (B) (C)** and **(D)** show the phenotypes of the egg, larva, pupa and adult of *H. armigera* (left panel) and *H. assulta* (right panel). **(E)** The green dashed line shows that the two black hair-base and stigma are in a straight line on the prothorax of *H. armigera* (left panel); the green dashes show that the two black hair-base and stigma are not in a straight line on the prothorax of *H. assulta* (right panel). The read arrows show the stigma of the prothorax. **(F)** The proleg crochet is in double order for *H. armigera* (left panel) but in single order for *H. assulta* (right panel). **(G)** Two anal spines of the abdominal end are born on a large black protuberance for the pupae of *H. armigera* (left panel); the two anal spines of the abdominal end do not share the black protuberance in *H. assulta* (right panel). **(H)** Forewing without white stripes, and the outer edge of the hindwing with a wide brown belt of *H. armigera* (left panel); forewing with a white stripe, and the outer edge of the hindwing with a narrow brown belt of *H. assulta* (right panel). Scale bars = 0.1 mm.

The two insect species in genus Helicoverpa, Cotton bollworm (*H. armigera*) and oriental tobacco budworm (*H. assulta*), are important insect pests of crops in China. They have similar external morphology. Figure 1 shows some taxonomic characteristics to distinguish these two species. Their eggs, larvae and pupae look like nearly same (Figure 1A-C). Only under the microscope, according to some taxonomic characteristics they can be distinguished (Figure 1E-G). The adult is the easiest stage to be distinguished by entomologist, because there are some special speckles and stripes on the wings (Figure 1D, H).

To show the relationship of *H. armigera* and *H. assulta*, multiple sequence alignments of spanning the 18S rRNA across 26 species from 23 orders (Additional file 1, Additional file 2: Figure S1A) and the expansion segment of the *COI* gene across 20 species of lepidopteran moths (Additional file 3, Additional file 2: Figure S1B) were constructed and supplied as Additional files. The results provide clues about the evolutionary origin of the phytophagous Noctuidae. The sister group of *H. assulta* and *H. armigera* is clustered on a clade. Some hypotheses on the sister group relatedness based on morphology are concordant with our molecular results.

### Sequencing of DGE-tag libraries and unique tag annotation

DGE-tag profile libraries were constructed from total RNA of *H. armigera* and *H. assulta* for four development stages (embryo, larva, pupa and adult). The summary sequence results are shown in Table 1. Low frequency tags were discounted under the assumption that many could have arisen through sequencing errors such as base substitution, deletion or addition at a single position [14]. Therefore, after eliminating low quality tags (containing Ns), copy numbers less than two and adaptor sequences, the remaining reads were called clean tags, of which more than 50% were singletons (tags with count equal to 1), which is typically observed in SAGE experiments [15]. We obtained approximately 23 million 17nt clean tags from both insect species. Their total unique clean tag (Uni-tag) numbers were also similar at approximately 357,000 (Table 1). Unique tag-to-gene assignments were conducted for the four development stages of *H. armigera* and *H. assulta* using SOAPdenovo program just permitting 1 bp mismatch [16]. On average, more than 75% of the uni-tags of *H. armigera* were mapped on transcripts; however, only 64.5% uni-tags of *H. assulta* mapped on transcripts. The total numbers of transcripts or genes were 268,145 and 230,591 for *H. armigera* and *H. assulta*, respectively, among which the annotated transcripts or genes were 88,857 and 75,157, respectively (Table 1). The Illumina short-reads sequence of *H. armigera* and *H. assulta* were submitted to NCBI Sequence Read Archive under the accession number of SRR628282 and SRR620569, respectively.

**Table 1:** **DGE-tag unique clean tags and tags percentage that map to genes in the four developmental stages of *H. armigera *and *H. assulta***

		**Embryos**	**Larva**	**Pupa**	**Adult**	**Total**
Raw data	*H. armigera*	6,202,369	5,766,391	6,126,237	6,021,919	24,116,916
	*H. assulta*	6,072,324	6,161,560	6,195,186	5,876,709	24,305,779
Clean tags	*H. armigera*	6,058,338	5,646,953	5,998,625	5,891,808	23,595,724
	*H. assulta*	5,955,877	6,048,552	6,084,239	5,759,082	23,847,750
Unique clean tags (Uni-tags)	*H. armigera*	97,646	80,673	89,193	89,330	356,842
	*H. assulta*	93,672	88,171	88,711	86,860	357,414
Uni-tag mapping to gene	*H. armigera*	71,623	64,679	64,458	67,385	268,145
	*H. assulta*	61,518	60,287	55,503	53,283	230,591
Mapping tag ratio (%)	*H. armigera*	73.35%	80.17%	72.27%	75.43%	75.14%
	*H. assulta*	65.67%	68.37%	62.57%	61.34%	64.52%
Gene numbers of tag mapping	*H. armigera*	22,890	21,080	21,987	22,901	88,857
	*H. assulta*	19,106	19,006	18,340	18,705	75,157
Mapping gene ratio (%)	*H. armigera*	34.27%	31.56%	32.92%	34.29%	
	*H. assulta*	28.61%	28.46%	27.46%	28.01%	

**Figure 2:**
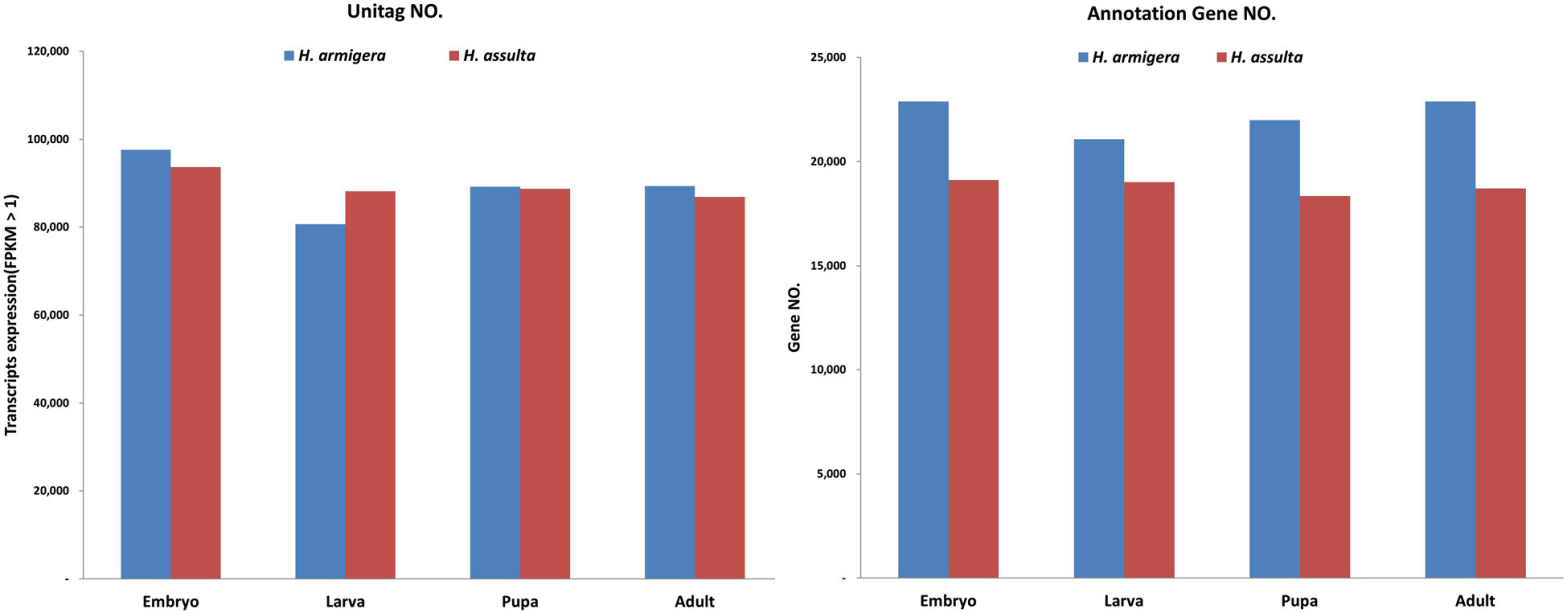
**Numbers of unique clean tags and identified genes of the four developmental stages in *H. armigera *and *H. assulta*. (A)** Numbers of unique clean tags. **(B)** Numbers of identified genes.

Although we obtained similar amounts of total unique clean tags from *H. armigera* and *H. assulta*, the numbers of uni-tags obtained from the different developmental stages in each insect were quite different (Table 1, Figure 2A). In theory, each uni-tag should be derived from one transcript [14]. According to this theory, the embryo stage has the highest number of transcripts compared with any other stages in the two species (97,646 and 93672 in *H. armigera* and *H. assulta*, respectively). Unexpectedly, the larval stage of *H. armigera* has the lowest number of transcripts, 80,673 (Table 1, Figure 2A). The gene annotation result indicated that 75.14% and 64.52% uni-tags of *H. armigera* and *H. assulta* corresponded to EST sequences in the *H. armigera* transcriptome library. The number of identified genes in *H. armigera* is higher than in *H. assulta*; however, the number of identified genes has no significantly difference at each developmental stage of the same species (Table 1, Figure 2B).

### Global analysis of differentially expressed genes between the two species

**Figure 3:**
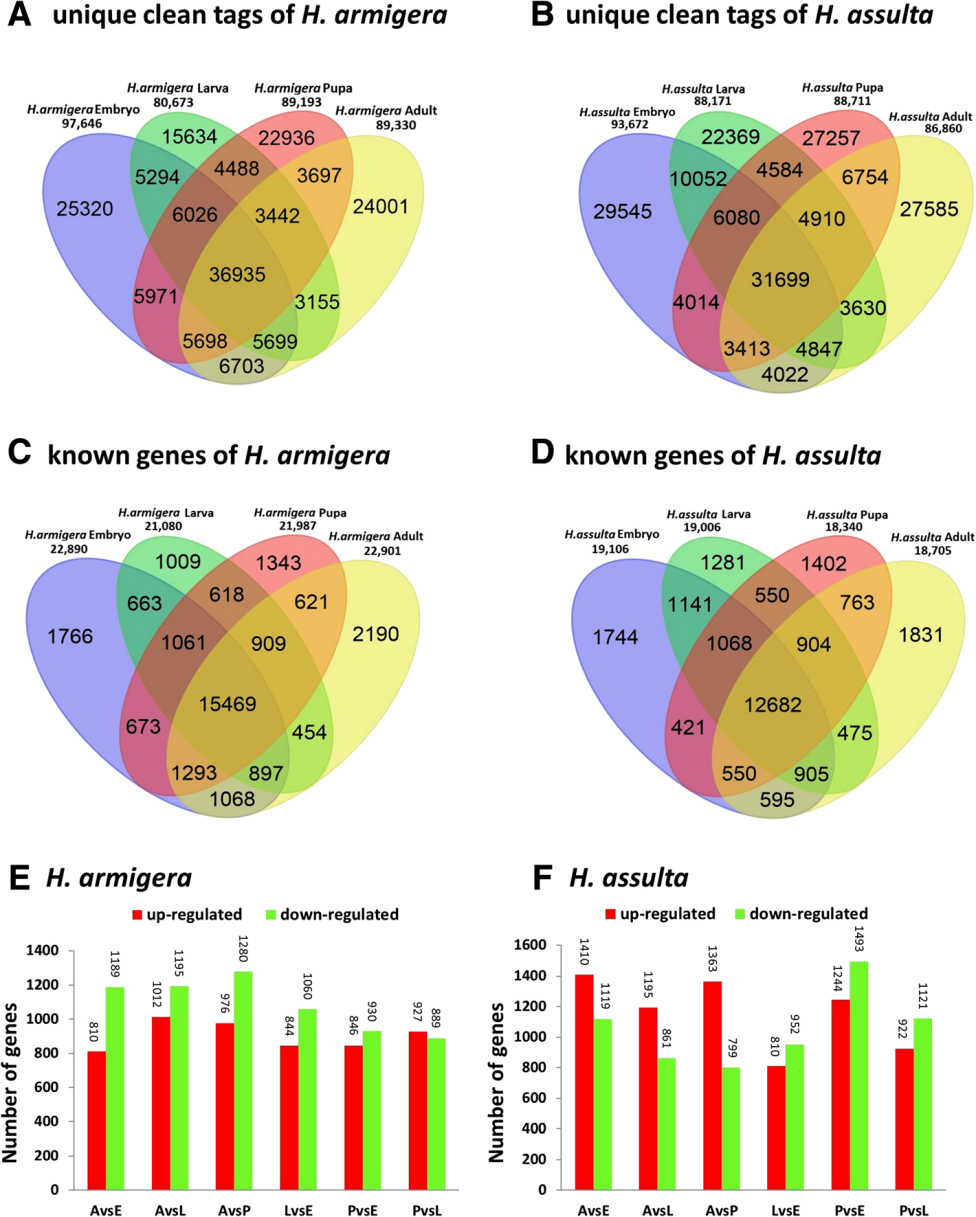
**Gene expression analysis of the *H. armigera *and *H. assulta. *(A)** and **(B)**: Venn diagram of unique clean tags showing the differential expression or coexpression between pairs developmental stages of *H. armigera* and *H. assulta*. **(C)** and **(D)**: Venn diagram of known genes showing the differential expression or coexpression between two to four developmental stages of *H. armigera* and *H. assulta*. **(E)**: Up- or downregulated expression of genes in *H. armigera*. **(F)**: Up- or downregulated expression genes in *H. assulta*. E: Embryo stage; L: Larval stage; P: Pupal stage; A: Adult stage.

The unique clean tags provide transcripts information for one species. Using a Venn diagram, developmental stage-specific transcripts and coexpressed transcripts between two to four developmental stages were shown between of *H. armigera* and *H. assulta* (Figure 3A, B). The analysis results revealed that the minimum numbers of coexpressed transcripts existed between the larval and adult stage in both *H. armigera* and *H. assulta* (3155 and 3630, respectively). This indicated that the biggest differences exist between these two stages among the four developmental stages. However, in *H. armigera*, the embryo and adult stage are probably “the closest neighbors” and have the most amount of 6703 coexpressed transcripts or genes. In *H. assulta*, the largest number of coexpressed transcripts was 10052 between the embryo and larvae stage. The uni-tag annotation results were also analyzed for differential expression and coexpressed transcripts or genes using a Venn diagram (Figure 3C, D).

The copy number of each unique tag provides quantitative information for the abundance of the transcripts or genes detected by the tags. Using the tag copy number, we can roughly estimate the expression level of each transcripts or gene. The dynamics of gene expression can be reflected by up- or down-regulation among the four development stages by pairwise comparisons (Figure 3E, F). Overall, the changes in gene expression levels between two developmental stages in *H. assulta* are more extreme than in *H. armigera*.

### Coexpressed transcripts or genes between two species

**Figure 4:**
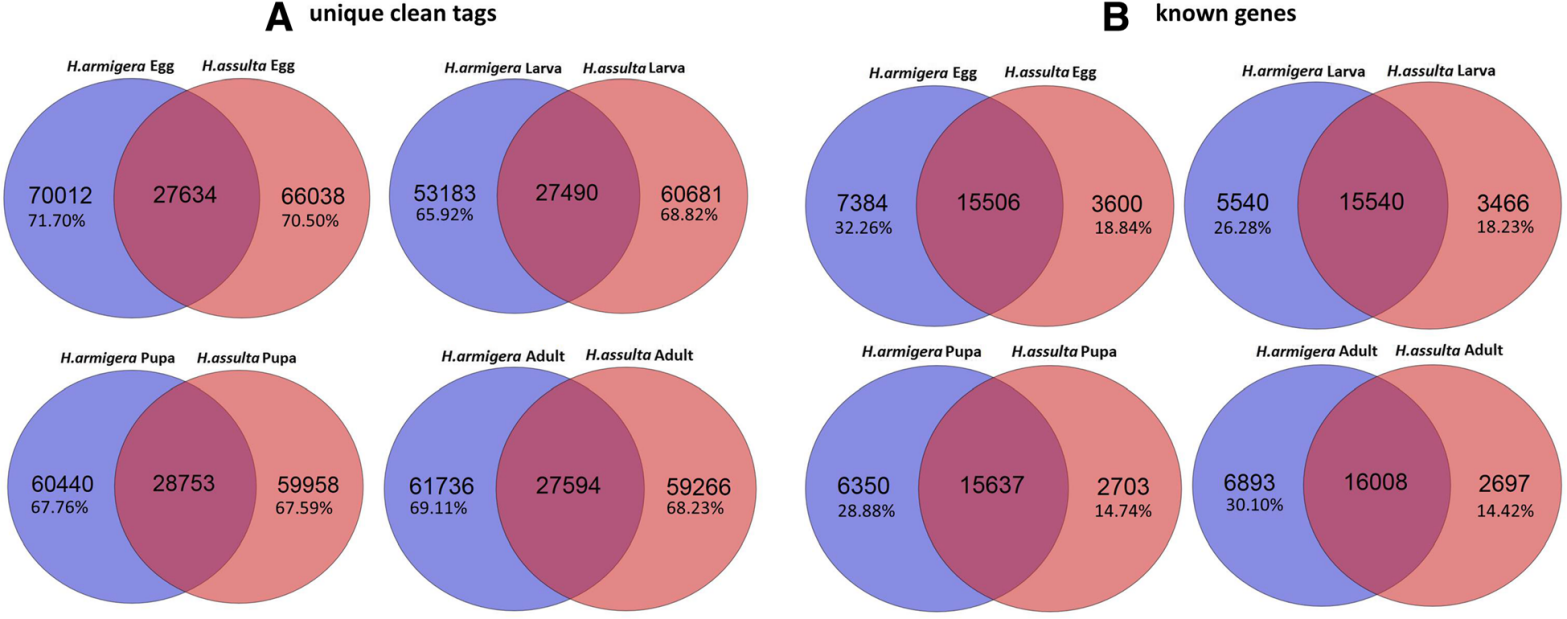
**Specific or coexpressed transcripts or known genes between the four developmental stages of *H. armigera *and *H. assulta*. (A)**: Unique clean tags indicate the potential transcripts; **(B)**: Known genes are the annotation results.

Comparing the unique clean tags between *H. armigera* and *H. assulta*, approximately 30% transcripts or genes are coexpressed in each developmental stage (Figure 4A, red region of overlap). This reflects the real situation of coexpression transcripts in these two insects. Because there are no whole genome annotation information for the two insect species, most of the differentially or coexpressed genes are unknown proteins, hypothetical proteins, enzymes or cytoskeletal proteins, which are annotated according to the *H. armigera* transcriptome results. In terms of annotated genes, about 67% to 85% genes in *H. armigera* and *H. assulta* are coexpression during the four developmental stages (Figure 4B, red region of overlap). GO analysis also confirmed that only in the larval stage there were more functional genes in *H. assulta* than in *H. armigera* (Additional file 4).

### Development and host range related transcripts or genes between two insect species

**Figure 5:**
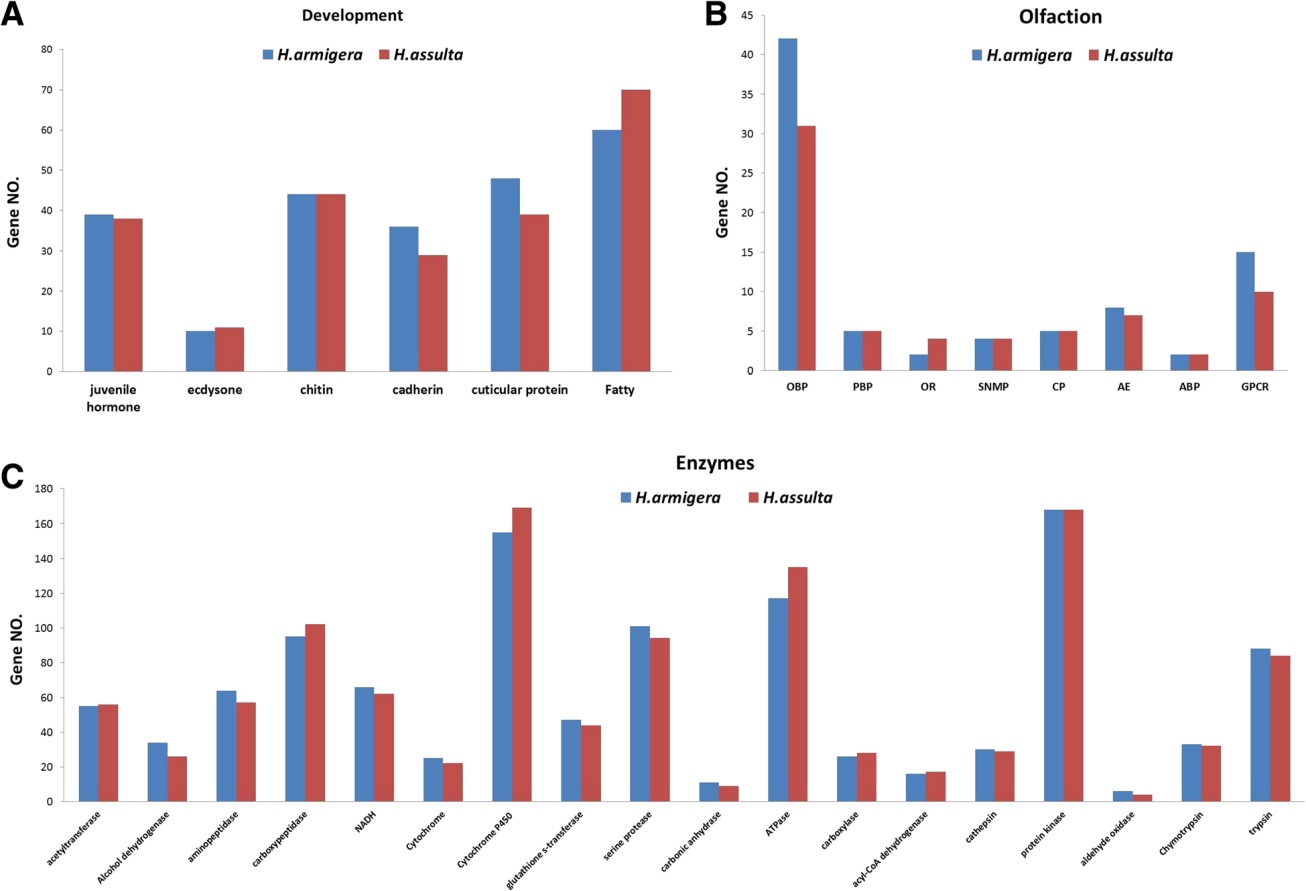
**Main categories of differentially expressed genes and transcripts between *H. armigera *and *H. assulta*. (A)** Growth and development related transcripts. **(B)** Olfaction related transcripts (OBP: odorant binding protein; PBP: pheromone-binding protein; OR: olfactory receptor; SNMP: Sensory neuron membrane protein; CP: chemosensory protein; AE: antennal esterase; ABP: antennal binding protein; GPCR: G protein-coupled receptor). **(C)** Transcripts for food digestion or detoxification related enzymes.

To explain why the two species have these similarities and differences, we focused on comparing the transcripts or genes that are related to growth and development, food digestion or detoxification enzymes, and host plant recognition. We identified 246 and 240 growth and development related transcripts, 1137 and 1138 transcripts for food digestion or detoxification related enzymes, and 83 and 68 olfaction related transcripts from *H. armigera* and *H. assulta*, respectively. The relative expression levels (by tag copy numbers) for these transcripts in each developmental stage of the two species and the annotation results are listed in Additional files 5, 6 and 7. In summary, the amounts of each type of transcript are similar between the two species (Figure 5A-C). The biggest difference in the number is odorant binding proteins (OBPs). There are 42 OBP transcripts in *H. armigera*, but only 31 in *H. assulta* (Figure 5B).

### Expression patterns of possible host-range-related transcripts or genes

**Figure 6:**
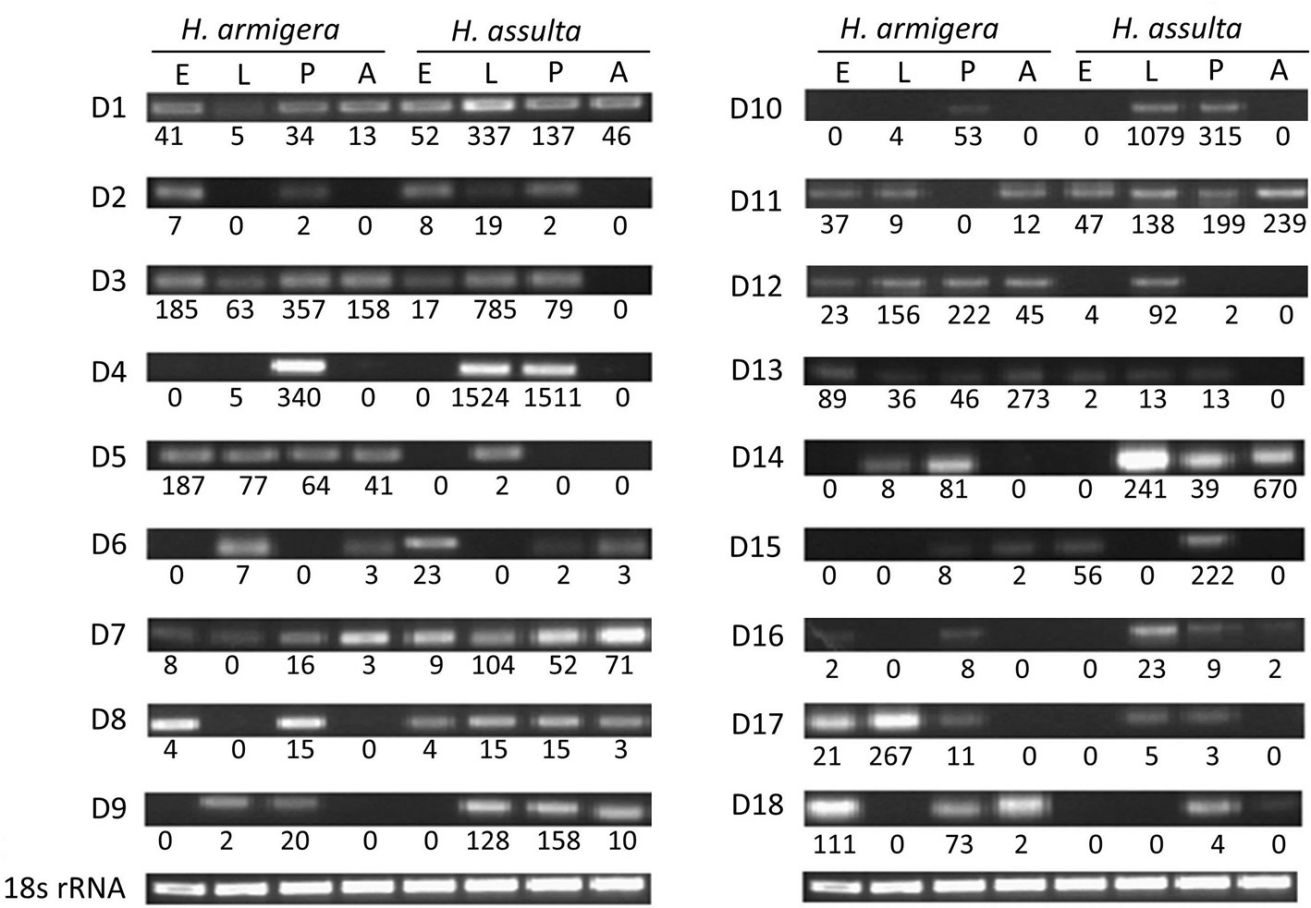
**Reverse transcription PCR results of different expression transcripts or genes.** E: Embryo stage; L: Larval stage; P: Pupal stage; A: Adult stage.

The expression patterns of transcripts can be divided into two categories by tag copy number among the four developmental stages between two species (Table 2). The first type is the similar expression pattern. For example, in Table 2, the Trypsin-1 gene just express at the embryo stage in the two species (Table 2, line 1). The Cytochrome C oxidase polypeptide III gene has nearly same high expression levels at all four development stages (Table 2, line 2). More information is shown in Additional files 5, 6, and 7. Genes and transcripts with similar expression patterns were not further analyzed in this study. The other expression pattern is showing a significant difference between two species. For example, the OBP3 gene is a carrier of odor molecules, which can protect odor molecules from enzymatic degradation [17,18]. Thus, OBP3 is likely to be an important gene in host plant selection. In this study, the expression pattern of OBP3 was significantly different among the four development stages between the two insect species (Table 2, line 5 D3). These kinds of transcripts or genes are probably the main reasons underlying the differences between the two species. The expressions of these types of transcripts or genes were confirmed by reverse transcription polymerase chain reaction (RT-PCR) (Figure 6). Most of expression patterns are nearly consistent with the digital expression tag copy number. These transcripts or genes should be further studied in host selection and adaptation.

**Table 2:** Expression patterns of food digestion or detoxification-related enzyme genes or transcripts according to tag copy numbers

**Pattern**	**Gene ID**	**seq_id**	**Embyro**		**Larva**		**Pupa**		**Adult**		**Annotation results**
			***H. armigera***	***H. assulta***	***H. armigera***	***H. assulta***	***H. armigera***	***H. assulta***	***H. armigera***	***H. assulta***	
*S1	P35035	HARM050658	74	71	0	0	0	0	0	0	Trypsin-1
S2	Q35826	HARM066614	234233	174746	185711	167262	159977	163095	132374	81008	Cytochrome c oxidase polypeptide III
D1	Q9VYY4	HARM012503	41	52	5	337	34	137	13	46	Cytochrome P450 4 g15
D2	P54191	HARM016746	7	8	0	19	2	2	0	0	Pheromone-binding protein-related protein 1
D3	AEB54582	HARM004409	185	17	63	785	357	79	158	0	OBP3
D4	Q00871	HARM066585	0	0	5	1524	340	1511	0	0	Chymotrypsin BI
D5	P46441	HARM051295	187	0	77	2	64	0	41	0	Putative ATPase N2B
D6	Q9VGG8	HARM002903	0	23	7	0	0	2	3	3	Probable G-protein coupled receptor Mth-like 5
D7	Q11001	HARM001041	8	9	0	104	16	52	3	71	Membrane alanyl aminopeptidase
D8	Q964T2	HARM001432	4	4	0	15	15	15	0	3	Cytochrome P450 9e2
D9	Q9QYZ9	HARM001863	0	0	2	128	20	158	0	10	Serine protease 30
D10	Q0II73	HARM066611	0	0	4	1079	53	315	0	0	Carboxypeptidase O
D11	P62333	HARM025783	37	47	9	138	0	199	12	239	26S protease regulatory subunit 10B
D12	O18598	HARM027201	23	4	156	92	222	2	45	0	Glutathione S-transferase
D13	Q9V675	HARM053132	89	2	36	13	46	13	273	0	Probable cytochrome P450 6 g2
D14	Q9V7G5	HARM039905	0	0	8	241	81	39	0	670	Probable cytochrome P450 4aa1
D15	P46430	HARM034436	0	56	0	0	8	222	2	0	Glutathione S-transferase 1
D16	ACV60230	HARM024407	2	0	0	23	8	9	0	2	antennal esterase CXE3
D17	Q27377	HARM028696	21	0	267	5	11	3	0	0	Putative odorant-binding protein A10
D18	AEX07273	HARM051397	111	3	0	0	73	4	2	0	odorant-binding protein

* S = similarity; D = difference.

## Conclusions

In this paper, we systematically analyzed the differences and similarities between the two sibling insect species, *H. armigera* and *H. assulta*. These characteristics make the two sibling species fitting for research on host range, evolution and pest control. By comparing the tag copy number of each developmental stage, we identified some differentially expressed transcripts or genes that are probably associated with host plant recognition and food digestion or detoxification. These genes provide important clues for further study.

SAGE is a method of large-scale gene expression analysis [19]. It is an ‘open’ system that permits the relative expression levels of almost all transcripts in an organism. DGE-tag profiles are a development of this technology using second-generation high throughput sequencing technology [20,21]. The DGE-tag profile results indicated that even between two sibling species, only 30% of transcripts are coexpressed in each developmental stage. The annotation results indicated that 67–85% genes are coexpressed in each developmental stage between the two species (Figure 4). These results further confirm that they are distinct two species at the genome level.

The unique clean tags number can provide quantitative information for the number of transcripts detected by tags. Using traditional SAGE technology, 50,000 to 100,000 tags could be collected, which represent 20,000 to 40,000 unique tags [15,19]. In this study, the high-throughput approach was adopted to implement the tag sequencing protocol on the Illumina platform [13]. Using this technology, we obtained more than 23 million clean tags from *H. armigera* and *H. assulta*, respectively. These data far more exceed the saturation requirements of sequencing [15,22]. The total unique clean tags were 356,842 and 357,414 for each species, which probably represent the total transcripts in the whole life cycle of the two insects. A total of 80,673 to 97,646 transcripts were identified for each developmental stage (Table 1, unique clean tags). These results indicated that the data sets were suitable for analyzing the differential expression of transcripts or genes.

In this study, we found that embryo stage expressed the most transcripts or genes in both species. Unexpectedly, the larval stage expressed the lowest numbers of transcripts in *H. armigera* (Figure 3A). Because the total clean tag number far more exceeded the saturation requirements, the difference is unlikely to be caused by sequencing bias. The most significant difference between the two insects is in their host ranges, which should be reflected at the larval stage.

Actually, the selection and adaptation of the host plant is decided by the insect’s internal factors and external environmental stimuli. When insects choose a plant as a host, they will be spawning and feeding, and then growth and reproduction on the plant. During this process, as external environmental factors, plant volatile odors are the most important cues for host plant selection. At the same time, as internal factors, insect OBPs not only can selectively bind certain types of odor molecules, but also can remove toxic substances and protect the odor molecules from enzymatic degradation [17,18]. Then, the downstream ORs (odorant receptors) will be activated, the chemical odor molecule information will be converted into an electrical signal, spread to the central nervous system, triggering an insect behavioral response [23-26]. In this research, we identified 42 and 31 OBP-related transcripts or genes from *H. armigera* and *H. assulta*, respectively (Figure 5B, Additional file 6: Table S5). This is the biggest difference among all the transcripts types. This is probably the main reason why these two sibling species have different host ranges. We also identified two and four OR-related transcripts or genes from *H. armigera* and *H. assulta*, respectively. These transcripts should be further studied to increase our understanding of the host range difference between the two species.

In addition to host selection, the other important aspect is host adaptation. During feeding, insect will inevitably swallow some poisonous secondary metabolites from plants. Therefore, insects have to develop an adaptation mechanism involving a series of detoxification enzymes [27,28]. These detoxification enzymes include cytochrome P450-dependent monooxygenases (P450s), glutathione-S-transferases and carboxylesterases (COEs) [27,29-32]. In this study, we found that there are more transcripts or genes for P450s, COEs and ATPases in *H. assulta* than in *H. armigera* (Figure 5C). GO analysis also confirmed that only in the larval stage there are more functional genes in *H. assulta* than in *H. armigera* (Additional file 4). Therefore, we suspected that because oriental tobacco budworm has a narrower host range, more detoxification enzymes would help them increase food detoxification and utilization efficiency. This should be further investigated in a future study.

The two species are also important agricultural insect pests. Considering all the similarities and differences between the two sibling species, we think they are a good model insect-pair for developing species-selective RNAi technology. Many studies have shown that RNAi is feasible technology in insect pest control [33-37]. The appeal of RNAi technology in pest control is that it is possible to design the pesticide to target only a single species or a group of related species, with minimal threat to other organisms [38]. To this end, it is necessary to identify species-specific target genes. The present study represents an effective strategy for identifying differentially expressed genes from related species that do not have genome sequences. Using DGE-tag profile technology, we identified many differentially expression transcripts or genes from the two sibling insect species. These genes not only provide clues for host range difference studies, but may also represent important targets for species-specific control by RNAi technology.

## Methods

### Insect culture and sample collection

*H. armigera* and *H. assulta* were originally obtained from Henan Agricultural University and maintained for several generations in our laboratory. They were fed an artificial diet at 25 ± 1°C under a light–dark cycle of 14:10 h. Moths were provided with 10% honey solution as food; larvae were fed on a modified artificial diet (wheat germ: 84.0 g; casein: 64.0 g; sucrose: 64.0 g; cellulose: 10.0 g; vitamin C: 10.0 g; Wechsler salt: 10.0 g; choline chloride: 3.0 g; sorbic acid: 3.0 g; nipagin: 3.0 g; agar: 30.0 g; vitamin complex: 8.0 g; vitamin C: 8.0 g; and water: 1000 mL). Thirty-four samples were collected from embryo to adult stages during the whole life cycle of *H. armigera* (1d, 2d, 3d embryos; one to six instar larvae; 1-7d pupae; and 1-9d adults (male and female separately)). The samples were immediately frozen in liquid nitrogen and stored at −80°C before RNA extraction.

### RNA isolation

Total RNA was isolated using a Qiagen RNA Extraction kit according to the manufacturer’s instructions. The RNA was treated with RNase-free DNase I for 30 min at 37°C (New England BioLabs) to remove residual DNA. Equivalent amounts of the 34 samples were merged into four pools of embryo, larva, pupa and adult. mRNA was isolated from DNA-free total RNA using a Dynabeads mRNA Purification Kit (Invitrogen).

### cDNA synthesis

Before cDNA synthesis, 5 μg total RNA was treated with RQ1 RNase-free DNase (Promega), according to the manufacturer's instructions, to ensure no DNA contamination. cDNA synthesis was then carried out with the purified RNA using the SuperScript III First-Strand Synthesis System (Invitrogen), following the manufacturer’s instructions. The RT reaction was performed using Mastercycler Gradient (Eppendorf). Briefly, 1 μg RNA, 50 μM oligo dT(20) and 10 mM dNTP mix were added together and incubated at 65°C for 5 min. The samples were then placed on ice for at least 1 min. After that, 2 μl 10 × RT buffer, 1 μl 25 mM MgCl2, 2 μl 0.1 M DTT, 40 U RNaseOUT and 200 U SuperScript III were added and incubation carried at 50°C for 50 min. The RT reaction was terminated by incubating at 85°C for 5 min and the residual RNA was removed by incubating at 37°C for 20 min with the addition of 1 μl RNaseH. The cDNA was stored at −20°C.

### Sequence tag preparation, sequencing and DGE-tag annotation

Sequence tags were prepared with Illumina’s Digital Gene Expression Tag Profiling Kit, according to the manufacturer’s protocol. A schematic overview of the procedure can be found in reference [39]. We extracted 6 μg total RNA, use Oligo(dT) magnetic beads adsorption to purify mRNA, and then use Oligo(dT) as primer to synthesize the first and second-strand cDNA. The 5' ends of tags can be generated by two types of Endonuclease: NlaIII or DpnII. Usually, the bead-bound cDNA is subsequently digested with restriction enzyme NlaIII, which recognizes and cuts off the CATG sites. The fragments apart from the 3' cDNA fragments connected to Oligo(dT) beads are washed away and the Illumina adaptor 1 is ligated to the sticky 5' end of the digested bead-bound cDNA fragments. The junction of Illumina adaptor 1 and CATG site is the recognition site of MmeI, which is a type of Endonuclease with separated recognition sites and digestion sites. It cuts at 17 bp downstream of the CATG site, producing tags with adaptor 1. After removing 3' fragments with magnetic beads precipitation, Illumina adaptor 2 is ligated to the 3' ends of tags, acquiring tags with different adaptors of both ends to form a tag library. After 15 cycles of linear PCR amplification, 95 bp fragments are purified by 6% TBE PAGE Gel electrophoresis.

After denaturation, the single-chain molecules are fixed onto the Illumina Sequencing Chip (flowcell). Each molecule grows into a single-molecule cluster sequencing template through Situ amplification. Then add in four types of nucleotides which are labeled by four colors, and perform sequencing with the method of sequencing by synthesis (SBS). Each tunnel will generate millions of raw reads with sequencing length of 35 bp. Image analysis and basecalling were performed using the Illumina Pipeline, where sequence tags were obtained after purity filtering. This was followed by sorting and counting the unique tags.

We filtered out low quality tags (containing Ns), copy number below 2 and adaptor sequences. Ultimately, ≈6 million clean sequence DGE-tags for each developmental stages of embryo, larva, pupa and adult were obtained. The DGE tags, which consist of the CATG restriction enzyme digested site and an additional 17 bp from each transcript, were *de novo* assembled using SOAPdenovo program just permitting 1 bp mismatch [16]. All of the tags were compared with the reference database of *H. armigera* cDNA library [40-42] and other insect nucleotide sequences (*Bombyx mori*, *Heliothis virescens*, *Spodoptera exigua*, *Prodenia litura* and *Manduca sexta*) from NCBI. The number of tags mapped on a transcript was used as a measure of the abundance of this transcript. The DGE-tag expression level was calculated by the RPKM (Reads Per kb per Million reads) method [43]. Functional annotation by Gene Orthology (GO, http://www.geneontology.org/) was run on Blast2go (http://www.blast2go.org/) [44]. The COG and KEGG pathway annotation were performed using Blastall online program against the Cluster of Orthologous Groups of proteins (COG, http://www.ncbi.nlm.gov/COG) and Kyoto Encyclopedia of Genes and Genomes (KEGG, http://www.genome.jp/kegg) databases, respectively. Briefly, sequences were searched against GenBank non-redundant database (Nr) with BLASTx algorithm [44]. The blast results were mapped to gene ontology terms and annotation was carried out using default annotation parameters in the Blast2Go software suit [44-46]. For further functional annotation, the KEGG mapping was carried out in Blast2Go.

### Semi-quantitative RT-PCR

The cDNA was amplified in a 50-μl reaction mixture containing 50 mM KCl, 10 mM Tris–HCl, 2 mM MgCl2, 0.2 mM of each deoxyribonucleoside triphosphates, 0.4 μM primers (Additional file 8), and 1 U *Ex Taq* polymerase (TaKaRa Biotech) using hot-start PCR. The PCR reaction conditions are shown in Additional file 8. To confirm that the products were fragments of the target genes, the Beijing Genomics Institute sequenced the PCR products using the same set of primers. Furthermore, cDNA samples with the strongest amplification were serially diluted, and a close correlation between the amount of product and initial cDNA was seen after PCR analysis. Ten microliters of each PCR product was visualized by electrophoresis on a 1% agarose gel stained with ethidium bromide.

## Abbreviations

DGE-Tag: Digital gene expression tag profile; SAGE: Serial analysis of gene expression; Uni-tag: Unique clean tag; EST: Expressed sequence tag; NCBI: National center for biotechnology information; GO: Gene ontology; SQ-RT-PCR: Semi-quantitative reverse transcriptase polymerase chain reaction; ORs: Odorant receptors; OBPs: Odorant-binding proteins; P450s: Cytochrome P450-dependent monooxygenases; GSTs: Glutathione-S-transferases; COEs: Carboxylesterases.

## Competing interests

The authors declare that they have no competing interests.

## Authors’ contributions

HL analyzed data; HZ and RG collected the samples, designed the primers and performed RT-PCR test; XM and HL designed the experiments and wrote the manuscript. All authors read and approved the final manuscript.

## Supplementary Material

Additional file 1: Table S1 Sequence name of the 18S Ribosomal RNA Genes. Click here for file

Additional file 2: Figure S1 Phylogenetic tree of insects from 18S rRNA and the CoI gene to show the relationship of *H. armigera* and *H. assulta*. Click here for file

Additional file 3: Table S2 COI gene sequences name and gene ID. Click here for file

Additional file 4: Table S3 Growth and development related genes and transcripts. Click here for file

Additional file 5: Table S4 Olfaction related genes and transcripts. Click here for file

Additional file 6: Table S5 Food digestion or detoxification related enzymes. Click here for file

Additional file 7: Table S6 GO analysis results for all of the transcripts between the two species. Click here for file

Additional file 8: Table S7 Primer pairs for RT-PCR. Click here for file
