# Supplementary figures and images for: Identification of differential expression genes associated with host selection and adaptation between two sibling insect species by transcriptional profile analysis
Source: BMC Genomics. 2013 Aug 28;14:582. doi: 10.1186/1471-2164-14-582 (PMC3765734; doi:10.1186/1471-2164-14-582)

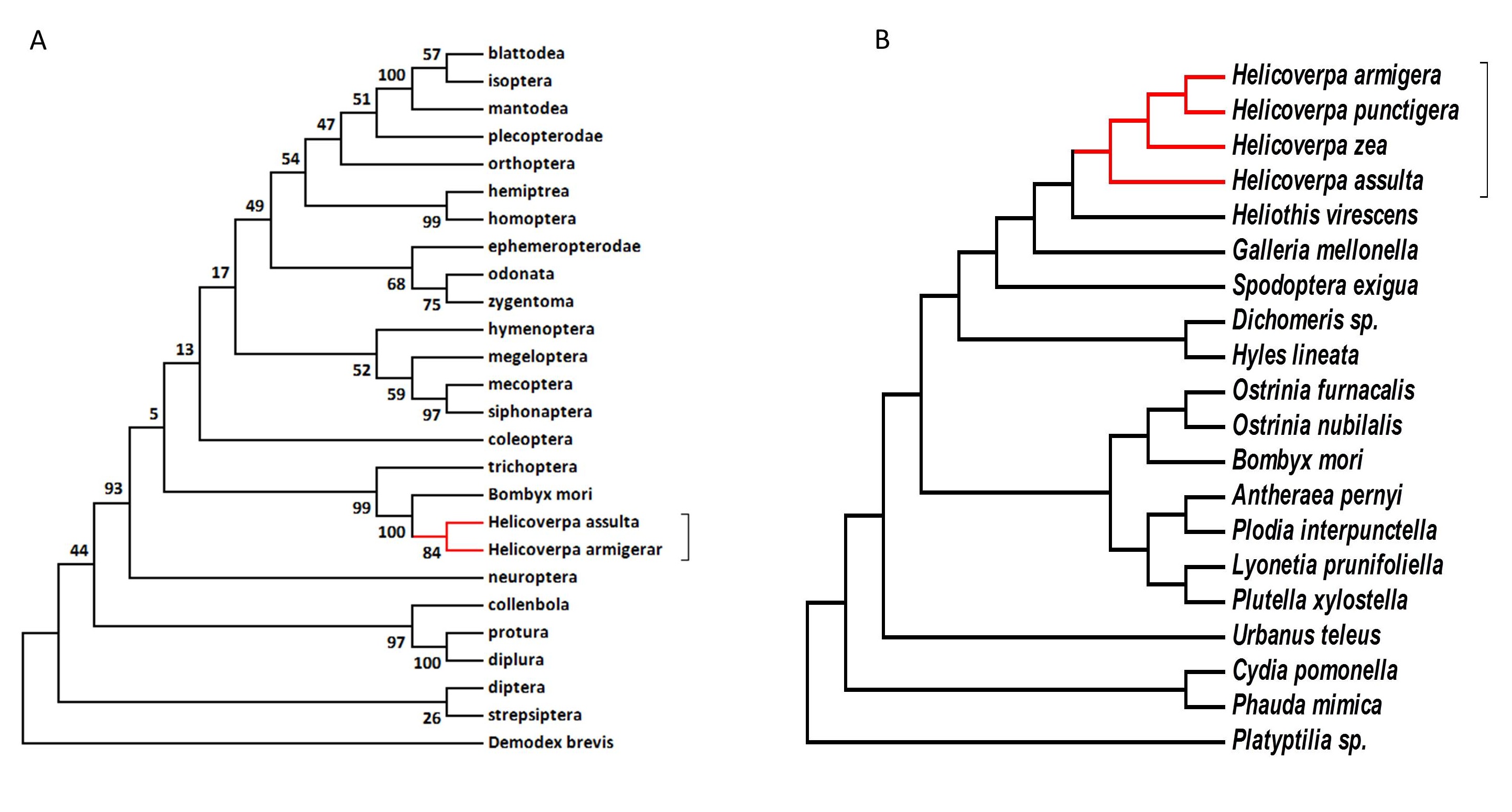

Supplement: Additional file 2: Figure S1 — Phylogenetic tree of insects from 18S rRNA and the CoI gene to show the relationship of H. armigera and H. assulta. [file 1471-2164-14-582-S2.jpeg]
